# Resistance-resistant antibacterial treatment strategies

**DOI:** 10.3389/frabi.2023.1093156

**Published:** 2023-01-30

**Authors:** Jonathan I. Batchelder, Patricia J. Hare, Wendy W. K. Mok

**Affiliations:** 1 Department of Molecular Biology and Biophysics, UConn Health, Farmington, CT, United States; 2 School of Dental Medicine, University of Connecticut, Farmington, CT, United States

**Keywords:** antibiotic resistance, evolutionary steering, mutation, stress response, combination therapies

## Abstract

Antibiotic resistance is a major danger to public health that threatens to claim the lives of millions of people per year within the next few decades. Years of necessary administration and excessive application of antibiotics have selected for strains that are resistant to many of our currently available treatments. Due to the high costs and difficulty of developing new antibiotics, the emergence of resistant bacteria is outpacing the introduction of new drugs to fight them. To overcome this problem, many researchers are focusing on developing antibacterial therapeutic strategies that are “resistance-resistant”—regimens that slow or stall resistance development in the targeted pathogens. In this mini review, we outline major examples of novel resistance-resistant therapeutic strategies. We discuss the use of compounds that reduce mutagenesis and thereby decrease the likelihood of resistance emergence. Then, we examine the effectiveness of antibiotic cycling and evolutionary steering, in which a bacterial population is forced by one antibiotic toward susceptibility to another antibiotic. We also consider combination therapies that aim to sabotage defensive mechanisms and eliminate potentially resistant pathogens by combining two antibiotics or combining an antibiotic with other therapeutics, such as antibodies or phages. Finally, we highlight promising future directions in this field, including the potential of applying machine learning and personalized medicine to fight antibiotic resistance emergence and out-maneuver adaptive pathogens.

## 1 Introduction

**FIGURE 1:**
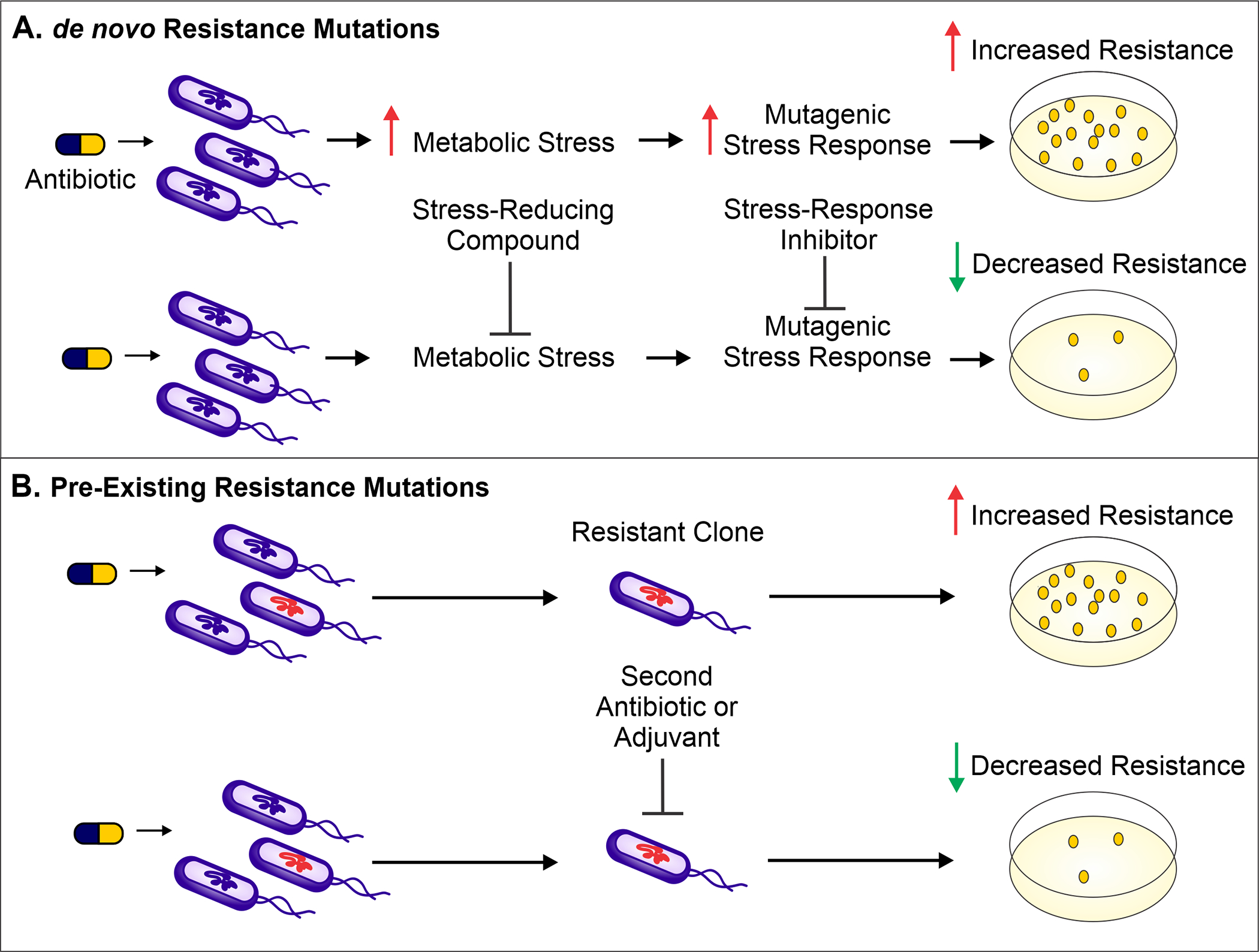
**(A)**. Antibiotic treatment increases metabolic stress (such as ROS) and leads to the activation of mutagenic stress-response pathways, resulting in *de novo* resistance mutations. The result is a higher number of antibiotic-resistant bacteria surviving antibiotic treatment (as shown on the antibiotic-containing media plates). To prevent these *de novo* resistance mutations and therefore limit resistance development, a compound can be administered to reduce the level of metabolic stress. Alternatively, mutagenesis can be reduced by inhibiting proteins important for activation of the mutagenic stress-response pathways. **(B)**. Antibiotic treatment selects for members of the population harboring pre-existing resistance-conferring mutations or genes. These resistant bacteria can be targeted by a second antibiotic or by using a variety of adjuvants—non-antibiotics that can be combined with antibiotic therapy to limit resistance development. Such adjuvants include enzyme or efflux pump inhibitors as well as bacteriophages that selectively target bacteria resistant to the antibiotic.

The use of antibiotics is central to the practice of modern medicine but is threatened by widespread antibiotic resistance (Centers for Disease Control and Prevention (U.S.), 2019). Antibiotics are a selective evolutionary pressure—they inhibit bacterial growth and viability, and antibiotic-treated bacteria are forced to either adapt and survive or succumb to treatment. The stress of antibiotic treatment can enhance bacterial mutagenesis leading to *de novo* resistance mutations (Figure 1A), promote the acquisition of horizontally transferred genetic elements that confer resistance, or trigger phenotypic responses that increase tolerance to drugs (Davies and Davies, 2010; Levin-Reisman et al., 2017; Bakkeren et al., 2019; Darby et al., 2022; ). Additionally, antibiotic treatment can select for the proliferation of pre-existing mutants already in the population (Figure 1B). Historically, the introduction of new clinical antibiotics has been closely followed by the identification of resistant bacterial isolates, making it clear that bacteria are remarkably capable of adapting to new treatments (Abraham and Chain, 1940; Davies and Davies, 2010). In this review, we describe therapies that inhibit the genetic changes underlying antibiotic resistance and target existing resistance mechanisms. These “resistance-resistant” strategies hold the key to preserving antibiotic efficacy for years to come.

## 2 Inhibiting bacterial evolvability to prevent antibiotic resistance development

The ability of bacteria to evolve in response to stresses, including those caused by antibiotics, is central to antibiotic resistance development. Therefore, limiting bacterial evolvability either by decreasing the stress causing the mutagenesis or by dampening the mutagenic response to the stressor is one approach to preventing *de novo* resistance mutations (Figure 1A).

### 2.1 Dampening mutagenic stressors

Antibiotic treatment can perturb bacterial metabolism and increase production of reactive metabolic byproducts (RMB), such as reactive oxygen species (ROS). These reactive metabolites are well known for their ability to damage macromolecules, such as DNA and proteins, and they can be mutagenic (Markkanen, 2017; Seixas et al., 2022; Wong et al., 2022). Although even sub-lethal doses of antibiotics are sufficient to increase mutagenesis that leads to increased minimum inhibitory concentrations, scavenging these reactive metabolites could prevent this effect (Kohanski et al., 2007; Kohanski et al., 2010).

Pribis and colleagues found that a small subpopulation of ciprofloxacin-treated *Escherichia coli* generate ROS which leads to mutagenic DNA repair and subsequent resistance development in this subpopulation (Shee et al., 2011; Pribis et al., 2019). To combat this problem, they treated *E. coli* with the antioxidant edaravone to reduce ROS levels, which ultimately reduced the number of resistance mutants that arose and preserved ciprofloxacin’s killing efficacy (Pribis et al., 2019). It must be acknowledged, however, that hindering RMB generation could reduce antibiotic efficacy. In contrast to the results of Pribis and colleagues, another group found that treating *E. coli* with RMB scavengers and ciprofloxacin, kanamycin, or mecillinam reduced the antibiotics’ killing effects (Wong et al., 2022). More research into the mechanisms of RMB involvement in antibiotic killing is necessary to determine whether prescribing antibiotics along with compounds to limit these stressors could be a valuable resistance-resistant strategy.

### 2.2 Inhibiting mutagenic stress responses

In contrast to lessening the stressors, bacterial evolvability can be reduced by inhibiting the evolution-driving pathways that respond to these stressors (Figure 1A) (Galhardo et al., 2007; Ragheb et al., 2019; Merrikh and Kohli, 2020). Given that many antibiotics can either directly (e.g., fluoroquinolones) or indirectly (e.g., antibiotics that stimulate RMB production) damage DNA integrity, one major pathway that allows bacteria to respond to antibiotic treatment and guard against deleterious DNA breaks is the SOS response (McKenzie et al., 2000; Maslowska et al., 2019; Podlesek and Bertok, 2020). This DNA repair process can involve the activation of mutagenic DNA polymerases that lack proofreading activity (Baharoglu and Mazel, 2014), potentially leading to resistance-conferring mutations (Podlesek and Bertok, 2020).

It has been hypothesized that SOS response inhibitors could limit adaptive mutagenesis and curb resistance development (McKenzie et al., 2000; Maslowska et al., 2019; Podlesek and Bertok, 2020). Cirz and colleagues found that SOS-deficient *E. coli* were unable to evolve resistance against ciprofloxacin or rifampicin (Cirz et al., 2005). Similarly, nanobodies or phages that prevent the cleavage of LexA, a repressor that undergoes proteolysis in response to DNA damage to induce the SOS response, block DNA repair and antibiotic resistance development (Lu and Collins, 2009; Maso et al., 2022). Other members of the SOS pathway have also been implicated as potential targets for inhibition along with antibiotic treatment in order to limit resistance mutations (Yakimov et al., 2021). Beyond targeting SOS response proteins, Ragheb and colleagues found that inhibiting Mfd, a DNA translocase found to increase mutagenesis and antibiotic-resistance development, could decrease the likelihood of resistance-conferring mutations (Ragheb et al., 2019). Although the regulation of enzymes responsible for mutagenesis varies across organisms, combining antibiotic therapy with the inhibition of conserved evolutionary drivers, like error-prone DNA polymerases, has immense potential to aid in the fight against antibiotic resistance development.

## 3 Evolutionary steering through sequential treatment and treatment cycling

**FIGURE 2:**
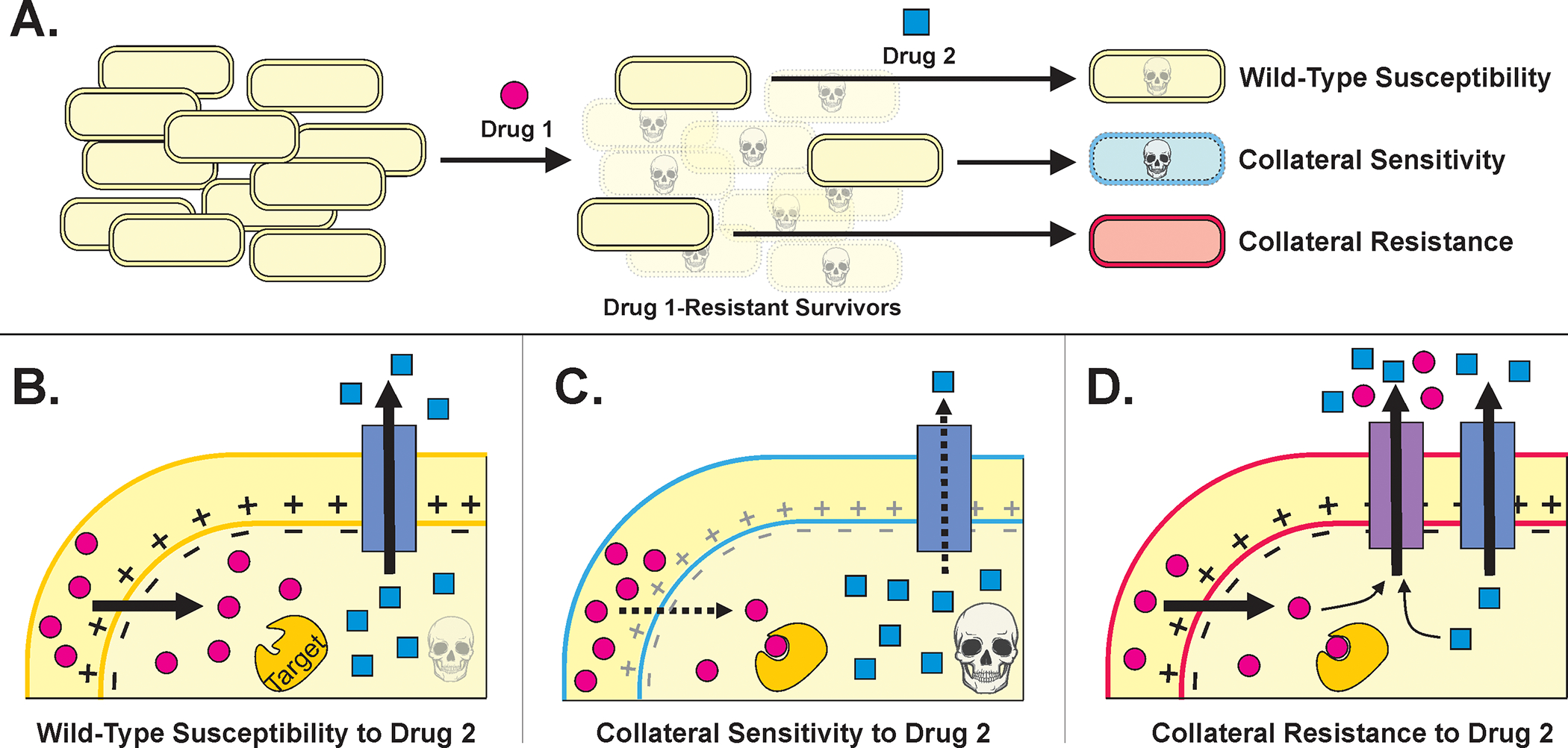
**(A)**.The strategies of treatment cycling and sequential treatment aim to capitalize on the collateral effects of resistance mutations. In this schematic, a population of cells undergoes a first treatment and then, to eradicate the resistant survivors, a second treatment is applied. Based on the mutations incurred during the first treatment, the cells may be more sensitive or resistant to the second treatment. **(B–D)**. Example mechanisms that may underly collateral effects for a Drug 1 whose uptake is proton motive force (PMF)-dependent and a Drug 2 that can be expelled from the cell via an endogenous efflux pump. (Figure adapted from Pál et al., 2015.) **(B)**. Cells that resist Drug 1 specifically—by mutating Drug 1’s target, perhaps—will have similar susceptibility to Drug 2 as wild-type cells. **(C)**. Cells that resist Drug 1 by decreasing PMF will also have decreased PMF-dependent efflux as a consequence, thereby increasing their sensitivity to Drug 2. **(D)**. Cells that resist Drug 1 by overexpressing or acquiring an efflux pump that can also expel Drug 2 will be collaterally resistant.

Developing antibiotic resistance often involves trade-offs for bacteria—resistant cells gain a fitness advantage in the presence of drug treatment but suffer a fitness defect when the selective pressure of the drug is removed (Lipsitch et al., 2000; Bergmiller et al., 2017). Furthermore, the genetic changes that a bacterium incurs to resist one drug can make it more susceptible or resistant to other antibiotics than the original wild-type cell in phenomena called collateral sensitivity and collateral resistance, respectively (Figure 2) (Szybalski and Bryson, 1952; Imamovic and Sommer, 2013; Lázár et al., 2013). Thus, subsequent administration of a different antibiotic—or adjuvant such as a bacteriophage (Chaudhry et al., 2017; Bull et al., 2019; Burmeister et al., 2020)—could exploit the resistant population’s weaknesses and lead to its eradication (Imamovic and Sommer, 2013; Kim et al., 2014; Roemhild et al., 2022).

Treatment cycling, also called sequential treatment, is a strategy that aims to capitalize on collateral sensitivities (Figure 2C). The goal is to kill the majority of bacteria with an initial antibiotic treatment and “trap” any bacteria that do develop resistance into collaterally sensitive genotypes, therefore making the second treatment more effective (Nichol et al., 2015; Nichol et al., 2019). Sequential treatment regimens have been used empirically in the clinic for decades, mainly in the context of switching from an intravenous antibiotic to a different oral antibiotic (Paladino et al., 1991; McMullan et al., 2016); but to our knowledge, the effectiveness of treatment cycling regimens in preventing clinical resistance has not yet been studied systematically.

Laboratory evolution experiments have shown that the success of a given cycling regimen depends on several factors, including the properties of each specific antibiotic, the duration of treatment before switching, a given bacterium’s genome, and the difficulty of acquiring a resistant genotype (Bal et al., 2010; Mouton et al., 2011; Chevereau et al., 2015; Baym et al., 2016; Barbosa et al., 2019; Batra et al., 2021). As an example of the latter factor, a single nucleotide mutation may be sufficient to confer resistance to an antibiotic like rifampicin, but other resistance genes, like acquisition of the tetracycline efflux pump, TetA, may require the opportunistic uptake of a larger genetic element (Perron et al., 2015). Furthermore, most of our understanding of bacterial responses to antibiotics is founded on *in vitro* experiments. In the host, antibiotic concentrations fluctuate between doses, and bacteria face additional selective pressures that have unknown consequences for their fitness and evolution (Yang et al., 2017; Manktelow et al., 2020). Altogether, these variables emphasize the fact that collateral effects are not guaranteed and must be considered probabilistic events that complicate the predictability of evolutionary trajectories (Nichol et al., 2019; Burmeister et al., 2020).

To ensure that treatment cycling is effective in practice, we need robust data sets and deep learning to predict the pleiotropic effects of resistance mutations and determine the least risky treatment regimens (Lopatkin and Collins, 2020; Anahtar et al., 2021). Collateral sensitivity has also been explored in the context of multidrug-resistant cancers, and advances from that body of research may provide insights on bacterial multidrug evolution, or vice versa (Vendramin et al., 2021). Alternatively, rather than switching antibiotics in sequence, there is evidence that adding a second antibiotic or administering combination therapies from the start might be more effective, as we discuss in the following section (D’Agata et al., 2008; Mouton et al., 2011; Barbosa et al., 2019; Angst et al., 2021).

## 4 Dual antibiotic therapy to prevent resistance emergence

### 4.1 Combining traditional antibiotics

Antibiotic treatment can select for pre-existing resistance mutations, but combining one antibiotic with another, or with an adjuvant that has a different target, can limit such selection (Figure 1B) (Wright, 2016). It is much less likely for a bacterium to simultaneously resist two treatments with different targets than it is to develop resistance toward monotherapy (Maron et al., 2022). Some examples of antibiotic combinations that have been shown to inhibit resistance development include ciprofloxacin plus amikacin against *Pseudomonas aeruginosa*, streptomycin plus para-aminosalicylic acid against *Mycobacterium tuberculosis*, and daptomycin plus rifampicin against methicillin-resistant *Staphylococcus aureus* (Bamberger et al., 1986; Fasching et al., 1987; Mouton, 1999; Garrigós et al., 2010; Mitchison and Davies, 2012; Worthington and Melander, 2013). Unfortunately, while many studies on antibiotic combinations focus on killing efficacy and synergy between combinations, few directly examine how these combinations affect resistance development (Deresinski, 2009; Samonis et al., 2012; Roemhild et al., 2022; Wang et al., 2022). Though counterintuitive, it has been shown that the combination of two antibiotics that suppress each other’s activity can actually hinder the development of resistance against either drug (Chait et al., 2007; Singh and Yeh, 2017; Liu et al., 2020). For example, the combination of daptomycin and rifampicin is less effective at killing *S. aureus* than treatment with either drug alone; however, this suppressive antibiotic combination prevents the emergence of rifampicin resistance because cells that do develop rifampicin resistance can resume growth and become more sensitive to daptomycin than the wild-type cells (Stein et al., 2016; Liu et al., 2020). These unexpected fitness dynamics indicate that the success of suppressive antibiotic combinations will hinge on the prevention of resistance evolution (Singh and Yeh, 2017; Liu et al., 2020).

### 4.2 Dual-mechanism compounds

Drawing inspiration from the potential success stemming from administering a combination of antibiotics, single compounds featuring two distinct antibacterial mechanisms that act synergistically have been developed. Several compounds have been developed in which two antibiotics (most commonly one fluoroquinolone and one DNA or protein synthesis inhibitor) are connected by either a cleavable or non-cleavable linker, and compounds based on these designs are currently undergoing clinical trials (Bremner et al., 2007; Pokrovskaya and Baasov, 2010; Ma and Lynch, 2016; Jubeh et al., 2020; Theuretzbacher, 2020). Additionally, Pentobra, which consists of the aminoglycoside tobramycin functionalized with an antimicrobial peptide, has been shown to better permeate bacterial membranes than tobramycin alone. This allows Pentobra to kill bacteria that normally resist aminoglycosides through poor uptake (Schmidt et al., 2015). Like studies of traditional antibiotic combinations, studies of these dual-mechanism compounds usually focus on killing efficacy rather than directly investigating their ability to prevent resistance development. Additionally, until recently, such two-pronged compounds often worked well against Gram-positive bacteria but failed against Gram-negative species (Gupta and Datta, 2019; Theuretzbacher, 2020). Excitingly, Irresistin-16 features one moiety that targets folate metabolism and another that targets membrane integrity; together, these moieties act to decrease survival of both Gram-negative and Gram-positive pathogens all while leaving no detectable resistant mutants (Martin et al., 2020).

## 5 Combining antibiotics with adjuvants

Instead of combining two antibiotics, an antibiotic can be combined with an adjuvant that has a different target or prevents selection for resistant bacteria. Aside from preventing *de novo* resistance mutations, another significant area of research is developing treatments that specifically address known resistance mechanisms such as antibiotic deactivating- or destroying-enzymes and multidrug efflux pumps (Amaral et al., 2014; Markley and Wencewicz, 2018). Additionally, combining traditional antibiotics with biologics such as antibodies and phages has shown great potential for preventing resistance development (Mariathasan and Tan, 2017; Torres-Barceló et al., 2022).

### 5.1 Counteracting enzymes that deactivate or destroy antibiotics

Some bacteria have enzymes that confer resistance by deactivating certain antibiotics (Blair et al., 2015; Wright, 2016; Markley and Wencewicz, 2018). While the antibiotic alone can kill members of the population lacking these enzymes, combining the antibiotic with inhibitors of these enzymes allows it to kill resistant bacteria as well (Wright, 2016; Liu et al., 2018; Markley and Wencewicz, 2018). The most successful example of this strategy is the combination of β-lactam antibiotics with β-lactamase inhibitors (Zhanel et al., 2013; Coleman et al., 2014; Brown and Wright, 2016; Wright, 2016). However, more research is needed before inhibitors of other antibiotic-inactivating enzymes can be used in the clinic. Park and colleagues found that anhydrotetracycline (aTc) binds to the active site of tetracycline destructases, thereby sparing some tetracycline from these enzymes. Treating *E. coli* expressing the tetracycline destructase Tet(56) with aTc significantly increased susceptibility to tetracycline (Park et al., 2017). Further research into the structures of these enzymes and the mechanisms by which they destroy or deactivate antibiotics may lead to the development of additional inhibitors that can preserve the efficacy of our antibiotic arsenal.

### 5.2 Inhibiting multidrug efflux pumps

In some cases, instead of deactivating or destroying an antibiotic, bacteria simply use efflux pumps to reduce the antibiotic’s intracellular concentration (Amaral et al., 2014; Li et al., 2015). Some efflux pump-encoding genes can be acquired through horizontal gene transfer, but several bacterial species naturally express a repertoire of efflux pumps that provide resistance to multiple antibiotics. Therefore, inhibiting efflux pumps to prevent the bacteria from escaping antibiotic attack is a promising strategy. Despite many compounds that are able to inhibit efflux pumps, this strategy has not been applied clinically because many of these inhibitors are toxic to humans (Ferrer-Espada et al., 2019). To combat this toxicity, Ferrer-Espada and colleagues found that antimicrobial peptides that increase the membrane permeability of *P. aeruginosa* increased the effectiveness of efflux pump inhibitors, which in turn increased the bacteria’s susceptibility to all antibiotics tested (Ferrer-Espada et al., 2019). This increased effectiveness should mean that a lower, and hopefully more clinically safe, dose of efflux pump inhibitor could be given to great effect. Additionally, it was recently reported that increasing the expression of multidrug efflux pump genes *acrAB* in *E. coli* reduces the expression of *mutS*, which is involved in DNA mismatch repair, and promotes spontaneous mutagenesis (El Meouche and Dunlop, 2018). These findings suggest that strategies to reduce *acrAB* expression can potentially limit mutagenesis and resistance development.

### 5.3 Antibody-antibiotic conjugates for enhanced targeting and efficacy

The conjugation of an antibiotic to a monoclonal antibody limits the number of bacteria that survive treatment compared to treatment with the antibiotic alone (Lehar et al., 2015; Kajihara et al., 2021; Cavaco et al., 2022). If fewer bacteria survive the treatment, then there are fewer bacteria left to develop and spread resistance (Bakkeren et al., 2019). This approach’s increased killing of the pathogen plus its ability to spare the microbiota from antibiotic exposure make it a promising resistance-resistant strategy. Often in an infection, pathogens are able to persist inside phagocytic or even non-phagocytic host cells while avoiding being digested (Helaine et al., 2010; Mariathasan and Tan, 2017; Peyrusson et al., 2020). In combined antibody-antibiotic therapy, an antibody carrying the antibiotic binds to a pathogen and opsonizes it for phagocytosis by immune cells. Once in the acidic internal environment of the phagocyte, the antibiotic will be cleaved off of the antibody and allowed to attack the pathogen, making it less able to persist inside the phagocyte (Dubowchik et al., 2002; Lehar et al., 2015; Cavaco et al., 2022). As mentioned before, a major benefit of this approach is that by targeting pathogens of interest with highly specific antibodies, the host microbiota should be spared from antibiotic exposure, reducing the likelihood of selecting for resistant mutants among commensal bacteria (Zurawski and McLendon, 2020). One pitfall of this approach is that designing and producing these conjugates remains very difficult because of the specific characteristics required for the antibody, antibiotic, and linker to each have maximum efficacy (Beck et al., 2017; Wei et al., 2018). Additionally, just as monoclonal antibodies designed to target pathogens for phagocytosis rely on at least partial immune system functionality (ter Meulen, 2007), antibody-drug conjugates may not be effective for immunocompromised patients.

### 5.4 Phage therapy to force pathogens toward antibiotic susceptibility

The combination of phage therapy with antibiotics has shown promise in reducing resistance development (Torres-Barceló et al., 2022). Bacteriophages specifically infect certain species or strains of bacteria and ultimately kill them through cell lysis (Lin et al., 2017). The range of bacteria that a given phage can infect is limited by the receptors available on the bacterial cell surfaces (de Jonge et al., 2019). With phage mining/hunting efforts and the development of modern genetic engineering techniques, the potential repertoire of phages that could be used against pathogens has greatly expanded (Chen et al., 2019; Gauthier et al., 2022). As with the other combination therapies mentioned, phage therapy and antibiotic therapy present two distinct selective pressures to the pathogen. Among the defenses that bacteria employ to resist being killed by phages (Egido et al., 2022), the most relevant are mutations that either remove or change the structure of the cell-surface receptor a given phage must bind in order to infect the bacterium. The result of this selection is that the population shifts toward decreased expression of the normal receptor on their surfaces. Then, if this receptor is important for the bacteria’s defense against antibiotics, their antibiotic susceptibility will be increased. In this way, phage therapy that targets surface proteins important for antibiotic resistance can shift the population toward antibiotic susceptibility and vice versa. For example, using phages that dock onto efflux pump components can select for mutants with defects such as nonfunctional multidrug efflux pumps, preventing them from expelling their usual antibiotic substrates (Chan et al., 2016; Gurney et al., 2020). Additionally, combined antibiotic-phage therapy can select for bacteria that only survive treatment by incurring mutations that reduce their fitness in the absence of treatment (Zhang and Buckling, 2012). Antibiotic-phage therapy has even shown promise in the clinic—an example is the successful treatment of a patient with an aortic arch prosthesis infected by *P. aeruginosa* (Chan et al., 2018). The phages targeted the multidrug efflux pumps of *P. aeruginosa*, making the pathogen more susceptible to treatment with ciprofloxacin and ceftazidime (Chan et al., 2018; Hatfull et al., 2022). These examples highlight the fascinating potential for phages and antibiotics together to prevent antibiotic resistance and effectively cure infections that are difficult to treat with either approach alone.

However, the use of bacteriophages has some limitations. First, unlike broad-spectrum antibiotics, many phages can only infect a few species or even a few strains of bacteria, making it difficult to effectively utilize phage therapy before identifying the infection’s causative agent (Loc-Carrillo and Abedon, 2011; Göller et al., 2021). This problem can be mitigated by prescribing a cocktail of several phages each with specificities for different bacteria (McVay et al., 2007; Kutateladze and Adamia, 2010; Loc-Carrillo and Abedon, 2011). Additionally, lysogenic phages integrate their DNA into the host’s genome and may transfer virulence or antibiotic-resistance genes to susceptible pathogens and commensal bacteria. Therefore, it is recommended that researchers focus on lytic phages that kill bacteria without integrating their DNA in order to prevent horizontal transfer of these harmful genes (Skurnik et al., 2007; Colomer-Lluch et al., 2011; Loc-Carrillo and Abedon, 2011). Finally, there are concerns that phages could cause unwanted immune responses. One study showed that phage therapy can increase serum levels of inflammatory markers (Oechslin et al., 2017; Liu et al., 2021). Additionally, the patient’s immune system can make antibodies against phages (Sunagar et al., 2010; Jun et al., 2014; Liu et al., 2021), but one study showed that production of such antibodies likely begins after the phages have already exerted their killing effects (Eskenazi et al., 2022). Phage therapy research has gained traction in the past decade after being largely overlooked in the 20^th^ century (de Jonge et al., 2019). Progress on this front can potentially illuminate strategies to improve the efficacy and overcome current limitations of phage therapy.

## 6 Future directions

We have discussed the promise as well as the challenges of several resistance-resistant therapeutic strategies including treatment cycling, combinations of two antibiotics, and combinations of antibiotic treatment with compounds that limit bacterial evolvability, resistance-conferring enzyme and efflux pump inhibitors, antibodies, and phages. In all, the goal of these strategies is to maximize killing of the pathogen population while minimizing resistance development. While these strategies have shown great promise in the lab, most of them require far more research before they can be applied in the clinic.

To overcome present limitations in formulating resistance-resistant therapeutic regimens, new technologies are being developed to improve informed treatment decision-making. For example, based on patient microbiomes and personal medical histories, machine learning algorithms are able to predict the likelihood of resistance development against a given antibiotic (Stracy et al., 2022). This approach may also predict horizontal gene transfer events from the host microbiome which can result in drug resistance (Andersson et al., 2020). Additionally, it has been suggested that shifting antibiotic-discovery pipelines toward more narrow-spectrum agents could help prevent resistance by sparing the microbiota and reducing selective pressure toward resistance development (Brown and Wright, 2016). There is also an exciting move toward rational drug design that anticipates resistance evolution and can hinder both wild-type and resistant cells (Manna et al., 2021). As we learn more about how antibiotic tolerant and persistent bacteria survive treatment and discover more efficacious methods of detecting these phenotypic variants in infections, we can leverage this information to select more appropriate combinations of therapeutics that can stall resistance evolution and achieve successful treatment outcomes (Singh and Yeh, 2017; Roemhild et al., 2022). Further research into optimizing the strategies outlined here in animal models and humans will help alleviate the antibiotic-resistance crisis and prevent many deaths due to antibiotic-resistant bacteria.
